# Supplementary material for: Neuronal Death Caused by HMGB1-Evoked via Inflammasomes from Thrombin-Activated Microglia Cells
Source: Int J Mol Sci. 2023 Aug 11;24(16):12664. doi: 10.3390/ijms241612664 (PMC10454604; doi:10.3390/ijms241612664)
Supplement: Supplementary file 1 [file ijms-24-12664-s001.zip › ijms-2447297-supplementary.pdf]

Supplementary data

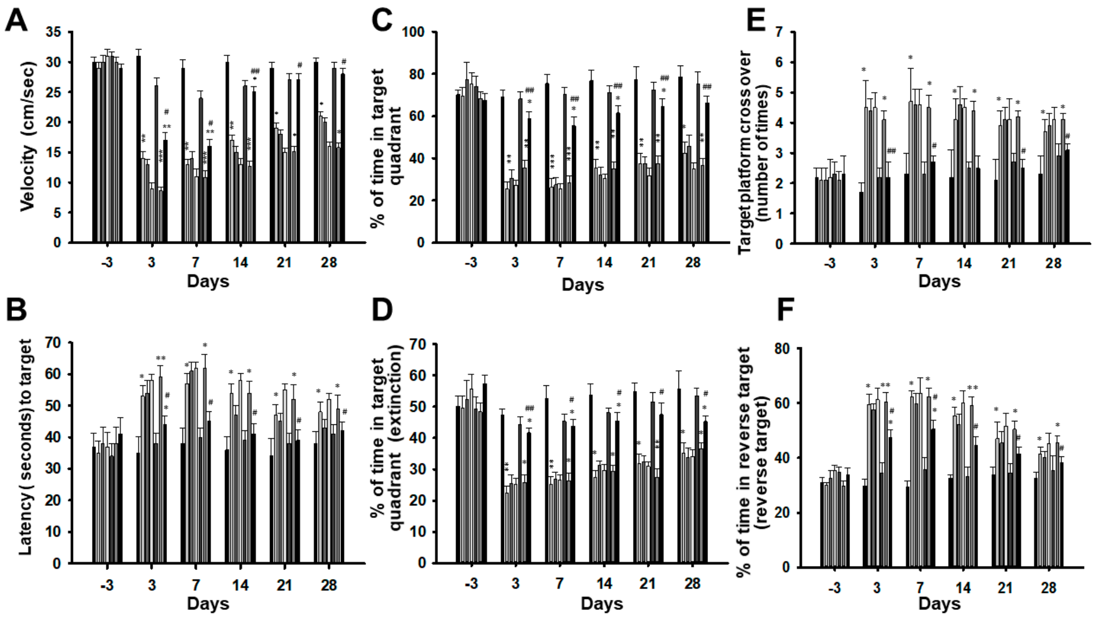

Supplementary Figure S1 *:* Representative water maze tests in different treatment groups shown in bar graph related to different time points (A) Quantitative analysis of swimming speed in different treatment group related to different time profiles (B) Quantitative analysis of swimming latency to the target in different treatment groups related to various time intervals (C) Quantitative analysis of percentage of time in quadrant target in different treatment groups related to various time profiles (D) Quantitative analysis of percentage of time in quadrant target at the extinction in different treatment groups related to various time profiles (E) Quantitative analysis of times of cross over to the target at the extinction in different treatment group related to various time profiles (F) Quantitative analysis of the percentage in the reverse target in different treatment groups related to various time profiles. The treatment group from left to right column was shown the sequene of PBS, L, T, T+L, HMGB1, T+ HMGB1, T+NA-HMGB1: see text. N=6, *: $p<0.05$, ** : $p<0.01$, and ***:$p<0.001$ indiacted the individual experimental groups related to PBS group. #:$p<0.05$ and ##:$p<0.01$ indicated the T+NA-HMGB1 group related to T group.

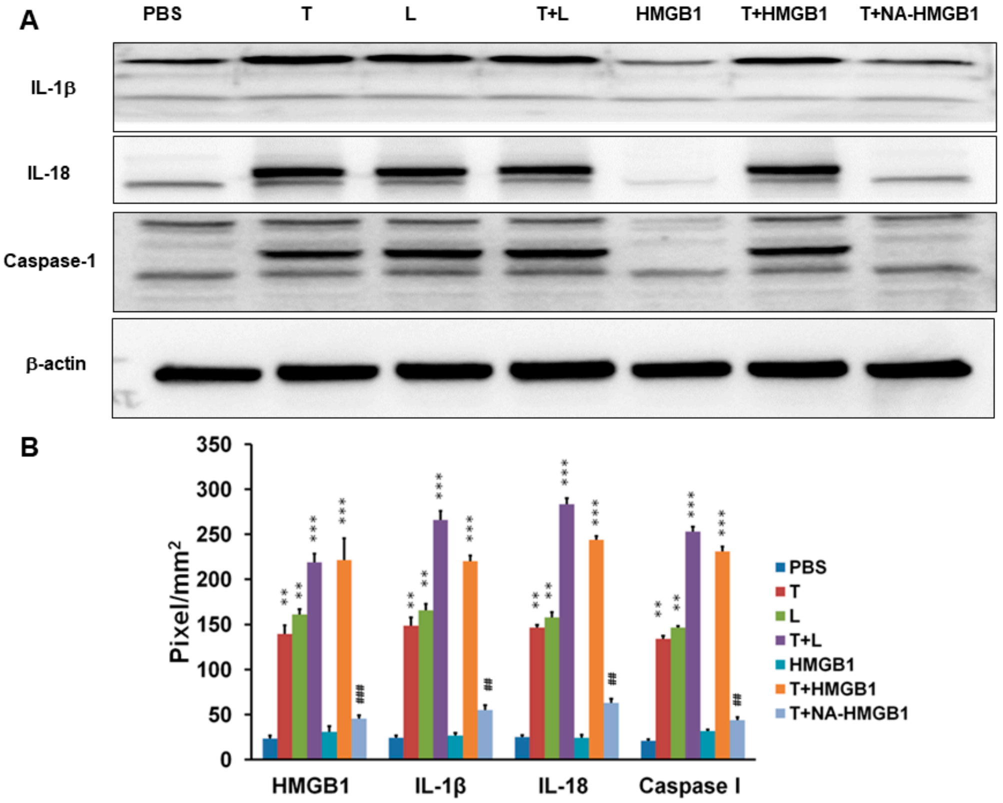

Supplementary Figure S2: The hippocampus tissue after different treatments analyzed by western blot and the intensity of immunohistochemistry staining 7 days after operation (A) Representative western blot analysis of the hippocampus in IL-1β, IL-18 and caspase I. β actin acted as an internal control. (B) Quantitative analysis of immunohistochemistry staining presented as pixel/ surface. N=6, *: $p<0.05$, **: $p<0.01$, and ***: $p<0.001$ indicated the individual experimental groups related to PBS group. ##: $p<0.01$ and ###: $p<0.001$ indicated the T+NA-HMGB1 group related to T group. T=thrombin; L=LPS; L+T=co-injection of LPS and thrombin; PBS, L, T, T+L, HMGB1, T+ HMGB1, T+NA-HMGB1: see text.

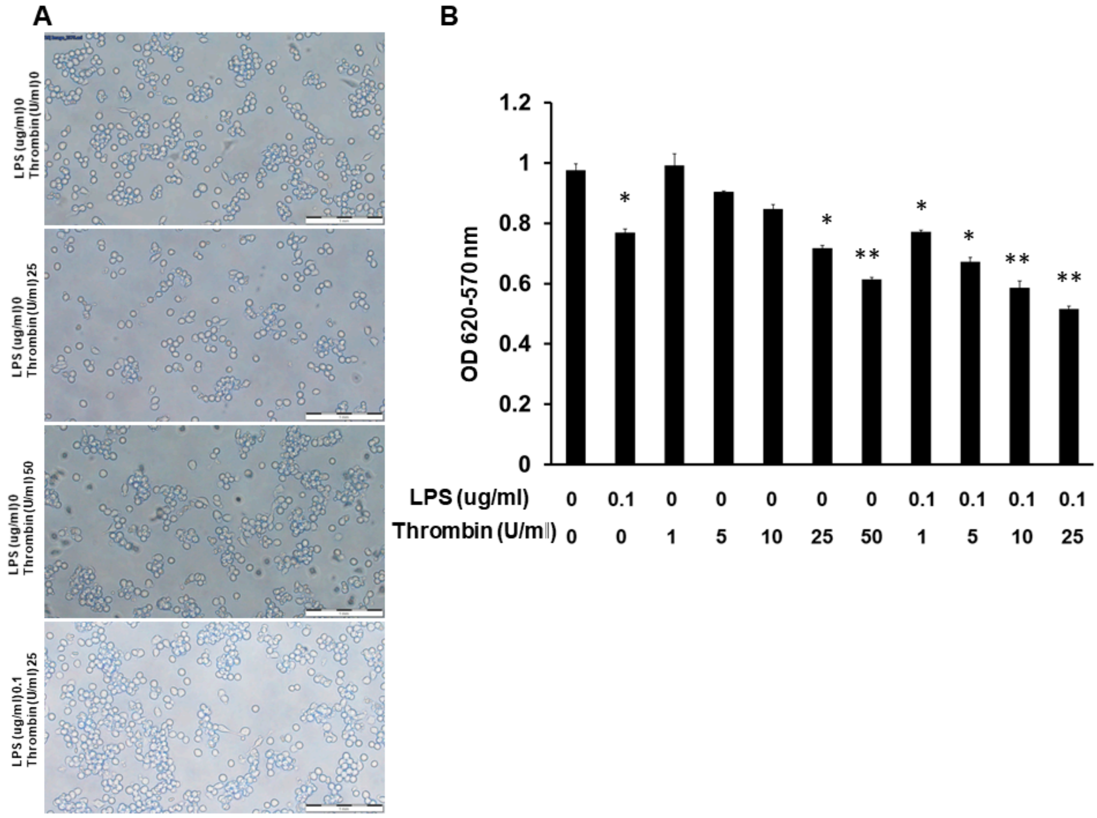

Supplementary Figure S3: The assessment of BV-2 cell viability under the combined treatment of LPS and Thrombin. BV-2 cells in a concentration of $1x10^5$ cells/mL were seeded in 96-well culture plates in a final volume of 100 μL/well subjected to the different combined treatment. Added to each well was a 10 μL solution containing 5 mg/mL 3-(4,5-Dimethyl-2-thiazolyl)-2,5-diphenyl-2H-tetrazoliumbromide (MTT), and incubation was at 37°C for 4 h. After discarding the supernatant and adding 100 μL DMSO, each well was analyzed with the ELISA reader at a wavelength of 570 nm. (A) The representative photograph of BV-2 cells in different treatment groups. Bar length=100μm. (B) The quantitative analysis of MTT assay in BV-2 microglia subjected to the combined treatment of LPS and thrombin. Each value is the mean± SEM of three samples. *: p <0.05; ** P<0.01, indicating a significant difference between experimental and control groups.
